# Supplementary material for: Ultrahigh Throughput Evolution of Tryptophan Synthase in Droplets via an Aptamer Sensor
Source: ACS Catal. 2024 Apr 10;14(8):6259–71. doi: 10.1021/acscatal.4c00230 (PMC11036396; doi:10.1021/acscatal.4c00230)
Supplement: Supplementary file 1 — cs4c00230_si_001.pdf [file cs4c00230_si_001.pdf]

Supplementary Information for

Ultrahigh throughput evolution of tryptophan synthase in droplets via an aptamer-sensor

Authors: Remkes A. Scheele[1], Yanik Weber[1], Friederike E. H. Nintzel[1], Michael Herger[1], Tomasz S. Kaminski[1,2] & Florian Hollfelder[1*]

Affiliations:

[1] Department of Biochemistry, University of Cambridge, Cambridge CB2 1GA, UK.

[2] Department of Molecular Biology, Institute of Biochemistry, Faculty of Biology, University of Warsaw, 02-096, Warsaw, Poland.

* Correspondence to fh111@cam.ac.uk, tel: +44 (0) 1223 766048.

This PDF file includes:

Supplementary Figure 1 – 11.

Supplementary Table 1.

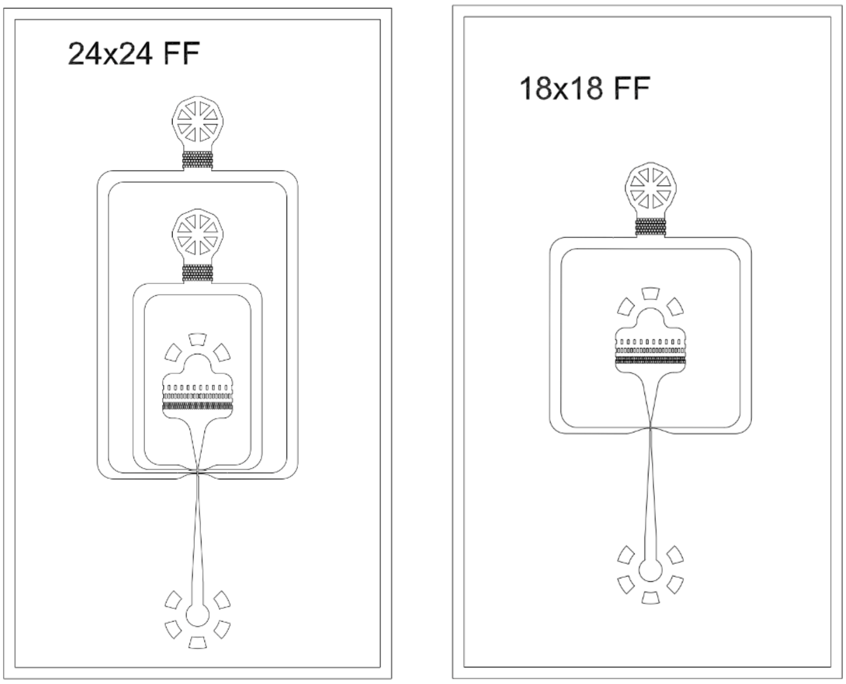

Supplementary Figure 1. *Chip designs for the first emulsification (left) and the second emulsification (right).* Files of these chip designs are available for download from our repository DropBase (https://openwetware.org/wiki/Dropbase:_Double-emulsion-02).

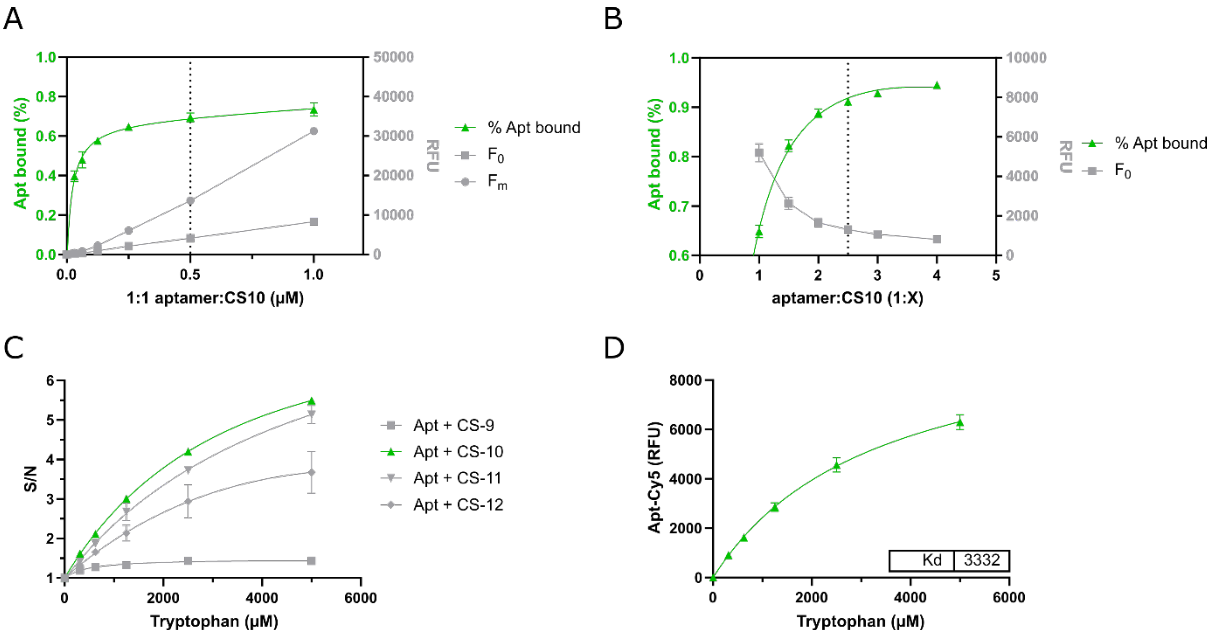

Supplementary Figure 2. *Optimisation of the Trp aptamer – CS10 sensor.* **(A)** The aptamer and the CS-10 were hybridised in a 1:1 ratio and measured at different concentrations of the duplex. F_0 (grey squares) denotes the (background) fluorescence of the Trp aptamer-CS10 complex when no Trp is present. F_m (grey circles) denotes the fluorescence of the Trp aptamer without CS-10 nor Trp is present. The % Apt bound was calculated by dividing the background fluorescence by the maximal fluorescence:

$$\%\ Apt\ bound = \left(1 - \frac{F_0}{F_m}\right) * 100\%$$

At lower concentrations, the percentage of CS-10 bound to the aptamer decreases, so that an increase in concentration reduces the background noise. The effect saturates around ~70% (0.5 µM aptamer:CS10 – dotted line), so these conditions were chosen for future experiments. **(B)** Trp aptamer (0.5 µM) was combined with different stoichiometries of CS-10. Again F_0 (grey squares) represents the (background) signal of the complex when no Trp is present. F_m is the same for all values, as 0.5 µM of aptamer was used for all stoichiometries and can be seen in panel 'A' at 0.5 µM. When 1x CS-10 is added, the background is the same as that observed in panel 'A', and roughly 70%. Increasing the number of equivalents of CS-10 reduces F_0 so that the percentage of Apt bound (see above) increases steadily. The effect saturated around 2.5 equivalents of CS-10 (dotted line), which was chosen for all future experiments. **(C)** Trp aptamer (0.5 µM final) was combined with CS (1.25 µM final) for all unique CSs, and hybridised. Trp was titrated to the mixtures, measuring the fluorescence (F_{Trp}). The signal-to-noise (S/N) was calculated by dividing the measured F_{Trp} at each concentration over the background noise (F_0) and plotted. **(D)** The raw titration curve of F_{Trp} for the CS-10 sensor was fitted to a Langmuir binding isotherm (Y = Bmax*X/(Kd + X) using Graphpad (Prism). The resulting K_{sens} value was found to be 3.3 mM. All experiments were performed in TAB (pH 7.4) at 25°C

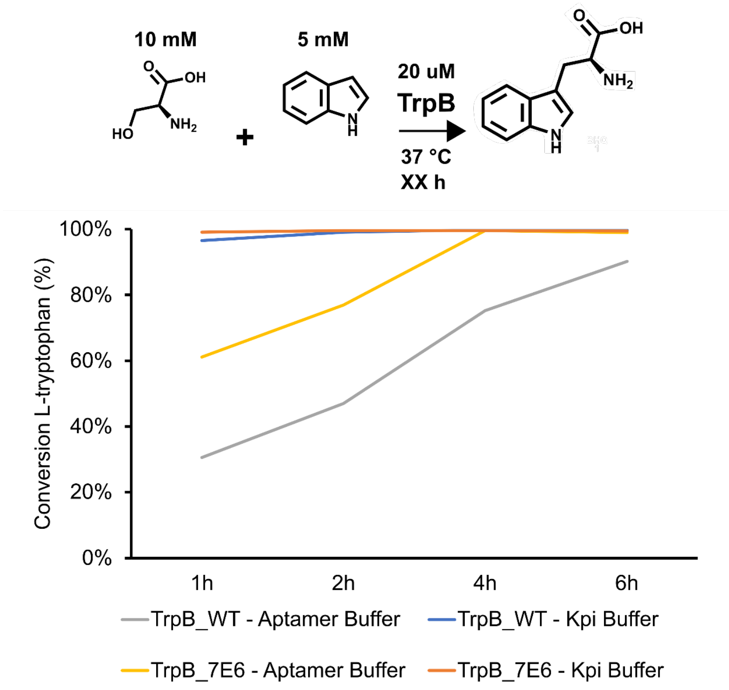

Supplementary Figure 3. The activity of TrpB in aptamer buffer and 50 mM KPi buffer. TrpB in 50 mM KPi (pH 8.0) or TAB (pH 7.4) was combined with substrates and left to react for one to six hours at 37°C (either in 50 mM KPi (pH 8.0) or TAB (pH 7.4) The reaction was quenched and analysed via HPLC to calculate the conversion to Trp displayed along the y-axis.

Table 1. Buffer optimisation of the aptamer sensor

Buffer	1	2	3	4	5	6	7	8	9
Na_2HPO_4[a]	0	10	10	10	10	10	10	10	10
KH_2PO_4[a]	50	2	2	2	2	2	2	2	2
KCl[a]	0	2.7	2.7	2.7	2.7	2.7	2.7	2.7	2.7
$MgCl_2$[a]	0	0	1	1	5	5	5	10	10
NaCl[a]	0	0	100	500	100	500	1000	500	1000
Tris-HCl[a]	50	0	0	0	0	0	0	0	0
EDTA[a]	1	0	0	0	0	0	0	0	0
pH	8	7.4	7.4	7.4	7.4	7.4	7.4	7.4	7.4

[a] All concentrations are in denoted in mM

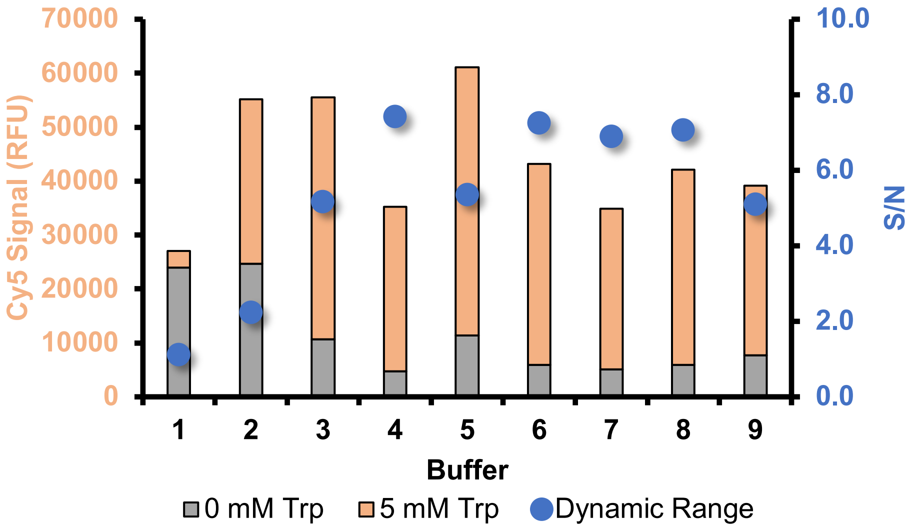

Supplementary Figure 4. Fluorescence of the aptamer sensor without Trp present (F_0, grey), with 5 mM Trp present (F_{Trp}, orange) and the S/N (F_{Trp}/F_0, blue circles) in ten different buffers. 6 = TAB, used throughout this study. All experiments were performed in TAB (pH 7.4) at 25°C

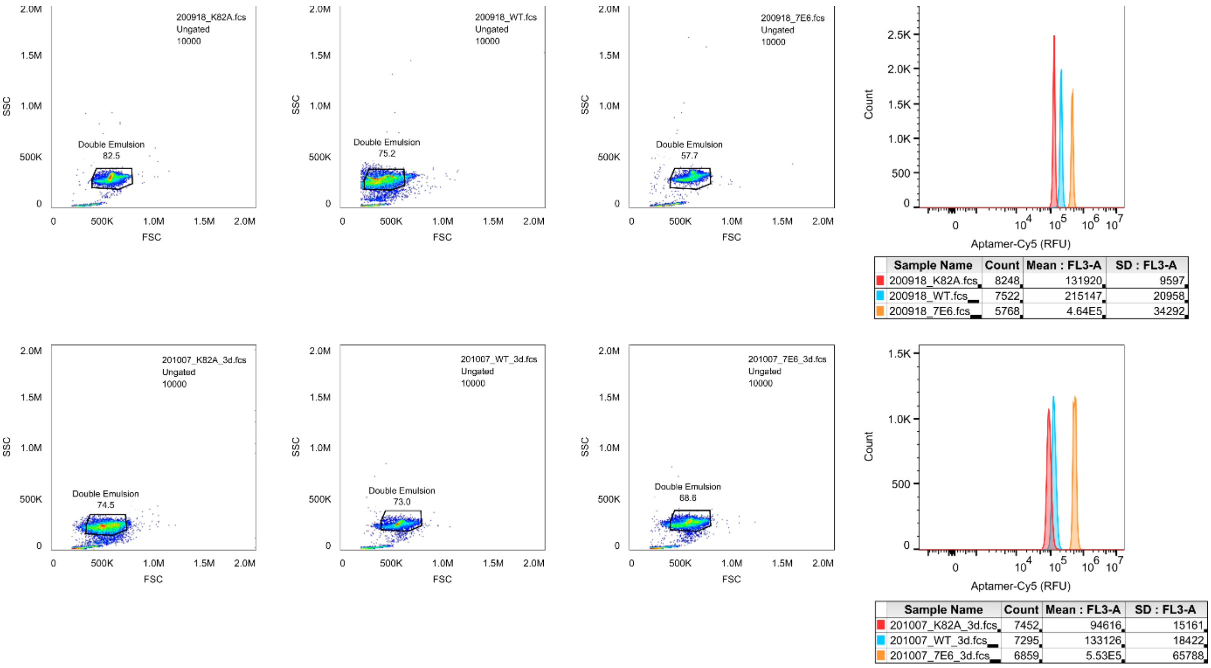

Sample Name	Count	Mean : FL3-A	SD : FL3-A
200918_K82A.fcs	8248	131920	9597
200918_WT.fcs	7522	215147	20958
200918_7E6.fcs	5768	4.64E5	34292

Sample Name	Count	Mean : FL3-A	SD : FL3-A
201007_K82A_3d.fcs	7452	94616	15161
201007_WT_3d.fcs	7295	133126	18422
201007_7E6_3d.fcs	6859	5.53E5	65788

Supplementary Figure 5. The activity of TrpB variants in double emulsion. Encapsulation of 20 µM TrpB, with 10 mM Ser and 5 mM indole with the Trp sensor in TAB (pH 7.4). The droplets were incubated for 72 hours at 37 °C before being encapsulated again and prepared for flow cytometry at RT. Flow cytometry analysis of FSC:SSC scatter plots for all six double emulsions (two for TrpB^{K82A}, two for TrpB^{WT}, two for TrpB^{7E6}). The double emulsion droplets were gated, analysing the average fluorescence in the Cy5 channel for all double emulsions (shown as a histogram), and the standard deviation. n = 10,000 sorting events for all scatter plots, and the respective numbers of double emulsion droplets *n* are shown in the table legend below the histograms. Double emulsion droplets were analysed on the CytoFLEX S (see methods).

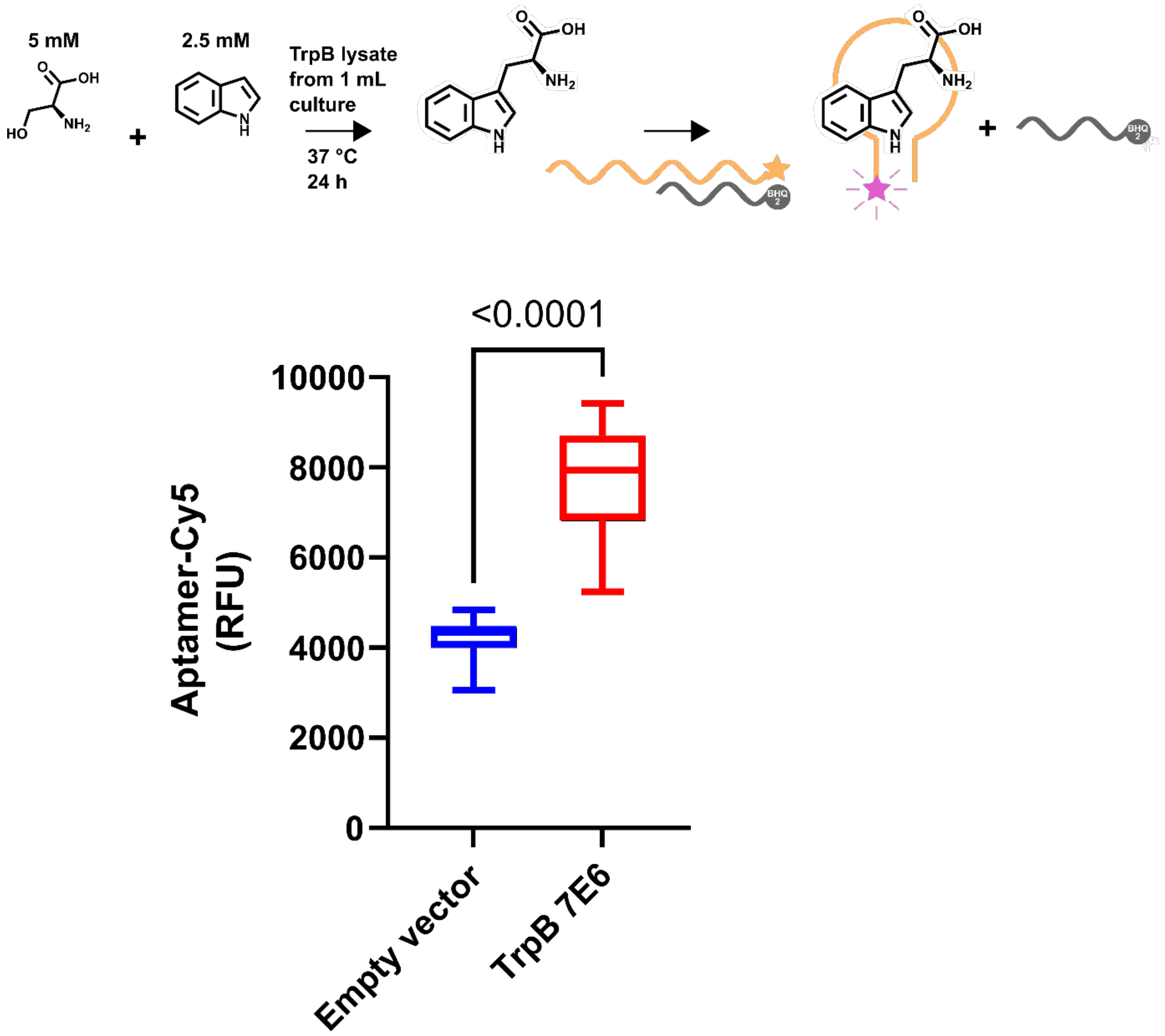

Supplementary Figure 6. Enrichment of active TrpB in plate-based format. Box and whisker plot shows 5th, 25th, 75th, 95th percentiles and the median for n= 32 screened wells. There was a significant difference between fluorescence measured in wells containing cells with empty vector or cells expressing $TrpB^{7E6}$ respectively (paired t-test, P value = <0.0001, n =32.). All wells containing $TrpB^{7E6}$ increased fluorescence compared to wells containing the empty vector control. Lysate from each well was combined with substrates in TAB (pH 7.4) overnight. After incubation, the mixture was cooled down to 25 °C, combined with the aptamer sensor in TAB (pH 7.4) and measured on UV-Vis after one hour for an endpoint measurement at 25°C.

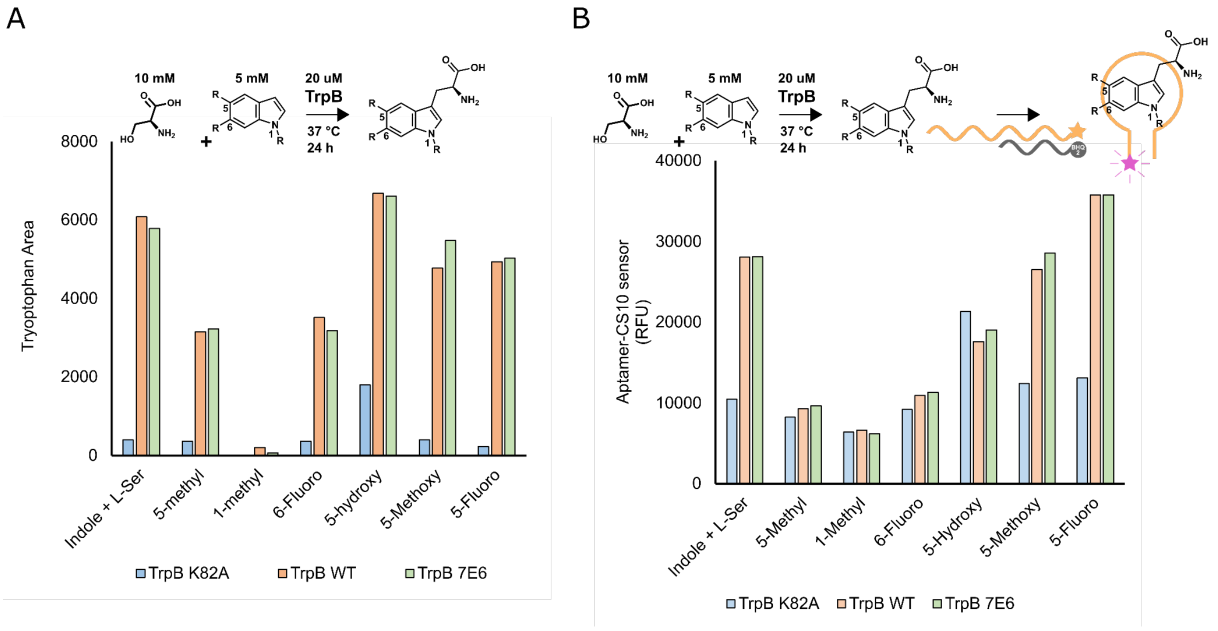

Supplementary Figure 7. Biosensing of Trp derivatives. (A) TrpB^{WT} and TrpB^{7E6} catalyse complete turnover of 5 mM Trp, 5-methyl-L-tryptophan, 6-fluoro-L-tryptophan, 5-hydroxy-L-tryptophan, 5-methoxy-L-tryptophan,and 5-fluoro-L-tryptophan from the respective indole substrates, whereas knock-out mutant TrpB^{K82A}, as expected, does not. Half of the reaction was quenched and analysed by HPLC in 'A', while the other half was combined with the Trp sensor in TAB, measuring fluorescence after 1h with UV-Vis in 'B'. **(B)** By comparing the fluorescent intensities of TrpB^{WT} and TrpB^{7E6} with TrpB^{K82A} we could determine whether the sensor is selective for the Trp derivative. The production of 5-methyl Trp, 6-fluoro Trp, and 5-hydroxy Trp by TrpB WT/TrpB^{7E6} could not be sensed by the aptamer sensor. The sensor was, however, capable of sensing both formation of 5-methoxy- and 5-fluoro-L-tryptophan, marking a ~2.5 to 3.5-fold difference in fluorescent intensity depending on whether active TrpB or inactive TrpB was added to the solution. All reactions were performed at 37°C in TAB (pH 7.4). After incubation, the mixture was cooled down to 25 °C, combined with the aptamer sensor in TAB (pH 7.4) and measured on UV-Vis after one hour for an end point measurement at 25°C.

Specificity of the aptamer sensor for Trp is of utmost importance – especially to differentiate between substrates (indole and Ser) and surface Trp (as part of TrpB) to the produced Trp (and highlighted by the lack of fluorescence for the K82A control with Indole and Ser present). However, versatility to other non-canonical amino acids which are not present in a cell and therefore should not cause background signal in directed evolution) is important as it allows for the screening of for value-adding compounds. We were excited to observe detection of 5-fluoro and 5-methoxy Trp. As the latter had issues dissolving, we opted to use 5-fluoro as the substrate for droplet screening.

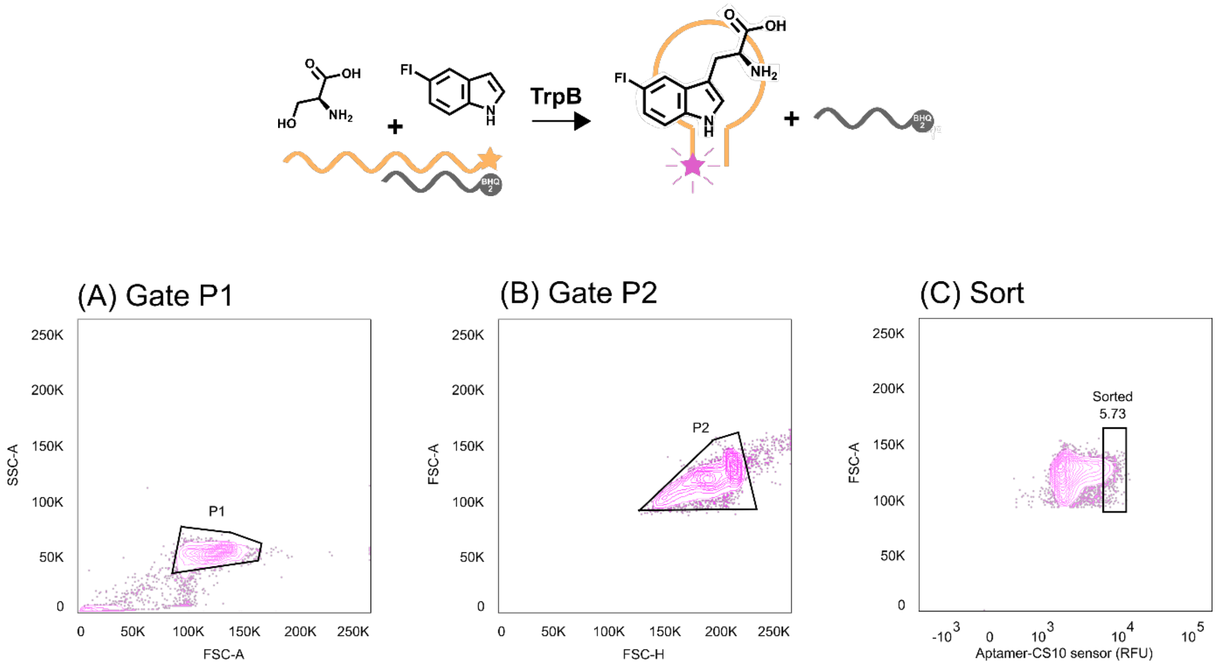

Figure	#Total DE analysed	P1 (%)*	P2(%)**	Sorted (%)***
A-C	233,699	88.6	97.8	5.8
*Of total number of DE. ** of P1. ***of P2.				

Supplementary Figure 8. Sorting of TrpB^{B10} library for 5-fluoro-L-tryptophan. Droplets were produced as described in the methods section and incubated at 37 °C after a 1 hour heat treatment @ 55°C. After formation of the double emulsion, droplets were sorted via FACS at RT. **(A)** Droplets were gated along gate P1 to distinguish droplets from debris. **(B)** Droplets were then gated along gate P2 to filter out the larger droplets resulting from a fusion of droplets. **(C)** The resulting droplet population was sorted according to Cy5-fluorescent intensities, collecting the top 5.7% for rescreening in plate-based format. Double emulsion droplets were sorted on an ARIA III flow cytometer (see Methods).

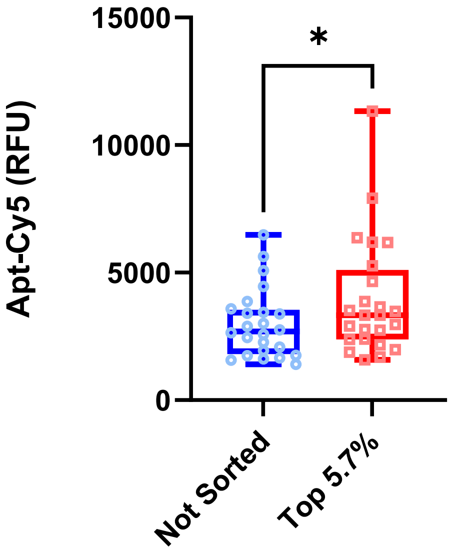

Supplementary Figure 9. Rescreening of individual variants in both the sorted and unsorted TrpB^{B10} library. The recovered genotype from both the unsorted fraction and the sorted fraction was transformed, and 24 individual colonies were picked and rescreened in plates for activity on 5-fluoroindole using the aptamer sensor. Lysate from each well was combined with substrates in TAB (pH 7.4) overnight. After incubation, the mixture was cooled down to 25 °C, combined with the aptamer sensor in TAB (pH 7.4) and measured on UV-Vis after one hour for an endpoint measurement at 25°C. The resulting fluorescence values, indicative of enzyme activity, are shown as individual dots in a box and whiskers plot. Box and whisker plot shows 5th, 25th, 75th, 95th percentiles and the median for n= 24 screened colonies. Star denotes siginicant difference (unpaired t-test, P value = 0.0436, n =24.)

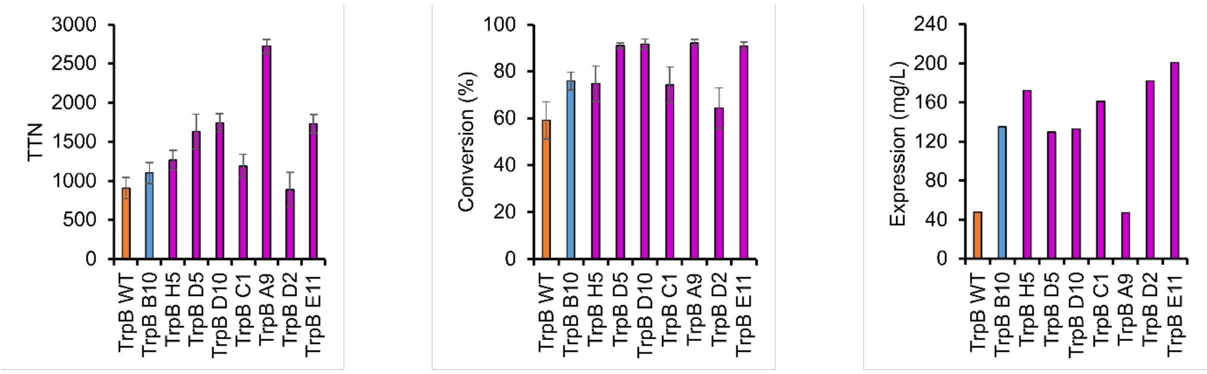

Mutant	Mutations
TrpB^{WT}	-
TrpB^{B10}	F176L
TrpB^{H5}	F176L, I68M, A214V
TrpB^{D5}	F176L, F35S, Y42F, I68L, D74E, Y128C, Q315R
TrpB^{D10}	F176L, G148C, F199Y
TrpB^{C1}	F176L, Y55C, K67N, A117V, Q210R , I235T, V248I
TrpB^{A9}	F176L, I102F, N166D, E364V, V368I
TrpB^{D2}	F176L, Y237F, V271D, R336H
TrpB^{E11}	F176L, A119E, I295N

Supplementary Figure 10. *Rescreening of individual variants of interest from the sorted fraction of the TrpB^{B10} library*. **(A)** Total turnover number (TTN) of all variants (max TTN = 10,000) on 5-fluoroindole after 14-hour hours incubation in TAB (pH 7.4) at 37°C. **(B)** Conversion of 5-fluoroindole (max TTN = 1000) after 14-hour hours incubation in TAB (pH 7.4) at 37°C. **(C)** Expression levels of each purified enzyme in mg/L.

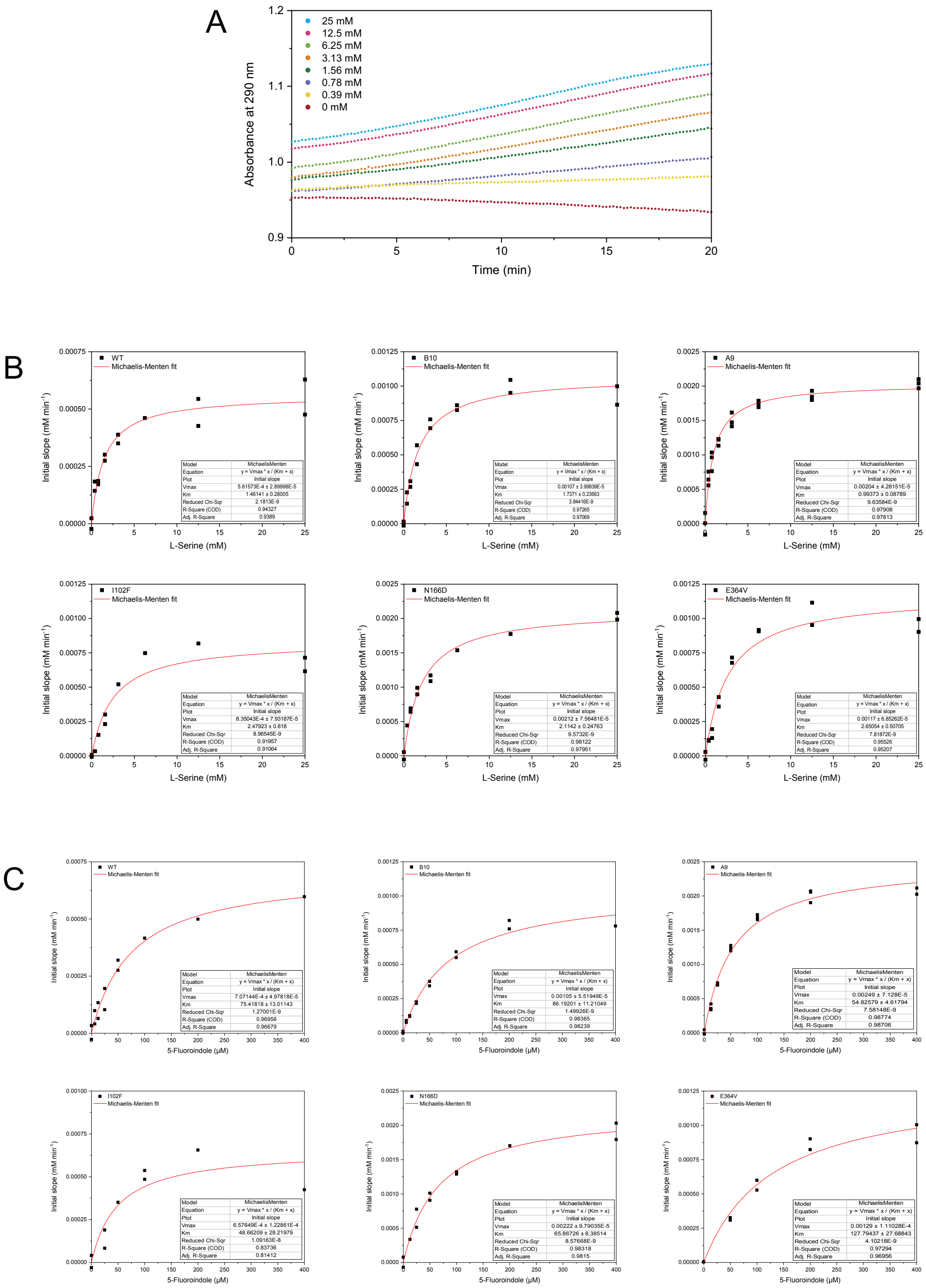

Supplementary Figure 11. *Michaelis-Menten kinetics of TrpB variants.* Kinetic measurements were performed at 37℃ by determining initial rates of 5-fluoro-tryptophan formation at 290 nm in a plate reader using a 96-well quartz plate. Fitting to Michaelis-Menten equation was conducted using *Origin 2018*. (A) Raw absorbance traces of one replicate of the L-serine kinetics for TrpB^{A9}. Experiments were conducted for 20 min and absorbance was measured every 10 seconds. Slopes were normalised on background absorbance changes and initial rates were calculated using a standard curve. (B) L-Serine kinetics were obtained with varying concentrations of Ser between 25 mM and 0.39 mM, 200 µM 5-fluoroindole, 20 µM PLP, and 10 µM TrpB in TAB at pH 7.4. (C) 5-Fluoroindole kinetics were obtained with varying concentrations of 5-fluoroindole between 400 µM and 6.25 µM, 25 mM Ser, 20 µM PLP, and 10 µM TrpB in aptamer buffer at pH 7.4.

Table 1. Sequences of TrpB variants used in this study.

TrpB

DNA sequence
ATGTGGTTCGGTGAATTTGGTGGTCAGTACGTGCCAGAAACGCTGATTGAACCCCTGAAAGAGCTGGAAA
AAGCTTACAAACGTTTCAAAGATGACGAAGAGTTCAATCGTCAGCTGAATTACTACCTGAAAACCTGGGCA
GGTCGTCCAACCCCACTGTACTACGCAAAACGCCTGACTGAAAAAATCGGTGGTGCTAAAATATACCTGA
AACGTGAAGACCTGGTTCACGGTGGTGCACACAAGACCAACAACGCCATCGGTCAGGCACTGCTGGCAA
AGTTTATGGGTAAAACTCGTCTGATCGCTGAGACCGGTGCTGGTCAGCACGGCGTAGCGACTGCAATGG
CTGGTGCACTGCTGGGCATGAAAGTGGACATTTACATGGGTGCTGAGGACGTAGAACGTCAGAAAATGA
ACGTATTCCGTATGAAGCTGCTGGGTGCAAACGTAATTCCAGTTAACTCCGGTTCTCGCACCCTGAAAGA
CGCAATCAACGAGGCTCTGCGTGATTGGGTGGCTACTTTTGAATACACCCACTACCTAATCGGTTCCGTG
GTCGGTCCACATCCGTATCCGACCATCGTTCGTGATTTTCAGTCTGTTATCGGTCGTGAGGCTAAAGCGC
AGATCCTGGAGGCTGAAGGTCAGCTGCCAGATGTAATCGTTGCTTGTGTTGGTGGTGGCTCTAACGCGA
TGGGTATCTTTTACCCGTTCGTGAACGACAAAAAAGTTAAGCTGGTTGGCGTTGAGGCTGGTGGTAAAGG
CCTGGAATCTGGTAAGCATTCCGCTAGCCTGAACGCAGGTCAGGTTGGTGTGTTTCATGGCATGCTGTCC
TACTTTCTGCAGGACGAAGAAGGTCAGATCAAACCAACCCACTCCATCGCACCAGGTCTGGATTATCCAG
GTGTTGGTCCAGAACACGCTTACCTGAAAAAAATTCAGCGTGCTGAATACGTGACAGTAACCGATGAAGA
AGCACTGAAAGCGTTCCATGAACTGAGCCGTACCGAAGGTATCATCCCAGCTCTGGAATCTGCGCATGCT
GTGGCTTACGCTATGAAACTGGCTAAGGAAATGTCTCGTGATGAGATCATCATCGTAAACCTGTCTGGTC
GTGGTGACAAAGACCTGGATATTGTCCTGAAAGTGTCTGGCAACGTGCTCGAGCACCACCACCACCACC
ACTGA

Translation
MWFGEFGGQYVPETLIEPLKELEKAYKRFKDDEEFNRQLNYYLKTWAGRPTPLYYAKRLTEKIGGAKIYLKRE
DLVHGGAHKTNNAIGQALLAKFMGKTRLIAETGAGQHGVATAMAGALLGMKVDIYMGAEDVERQKMNVFRM
KLLGANVIPVNSGSRTLKDAINEALRDWVATFEYTHYLIGSVVGPHPYPTIVRDFQSVIGREAKAQILEAEGQLP
DVIVACVGGGSNAMGIFYPFVNDKKVKLVGVEAGGKGLESGKHSASLNAGQVGVFHGMLSYFLQDEEGQIKP
THSIAPGLDYPGVGPEHAYLKKIQRAEYVTVTDEEALKAFHELSRTEGIIPALESAHAVAYAMKLAKEMSRDEIII
VNLSGRGDKDLDIVLKVSGNVLEHHHHHH-

TrpBHistag

TrpB_K82A

DNA sequence
ATGTGGTTCGGTGAATTTGGTGGTCAGTACGTGCCAGAAACGCTGATTGAACCCCTGAAAGAGCTGGAAA
AAGCTTACAAACGTTTCAAAGATGACGAAGAGTTCAATCGTCAGCTGAATTACTACCTGAAAACCTGGGCA
GGTCGTCCAACCCCACTGTACTACGCAAAACGCCTGACTGAAAAAATCGGTGGTGCTAAAATATACCTGA
AACGTGAAGACCTGGTTCACGGTGGTGCACACGCGACCAACAACGCCATCGGTCAGGCACTGCTGGCAA
AGTTTATGGGTAAAACTCGTCTGATCGCTGAGACCGGTGCTGGTCAGCACGGCGTAGCGACTGCAATGG
CTGGTGCACTGCTGGGCATGAAAGTGGACATTTACATGGGTGCTGAGGACGTAGAACGTCAGAAAATGA
ACGTATTCCGTATGAAGCTGCTGGGTGCAAACGTAATTCCAGTTAACTCCGGTTCTCGCACCCTGAAAGA
CGCAATCAACGAGGCTCTGCGTGATTGGGTGGCTACTTTTGAATACACCCACTACCTAATCGGTTCCGTG
GTCGGTCCACATCCGTATCCGACCATCGTTCGTGATTTTCAGTCTGTTATCGGTCGTGAGGCTAAAGCGC
AGATCCTGGAGGCTGAAGGTCAGCTGCCAGATGTAATCGTTGCTTGTGTTGGTGGTGGCTCTAACGCGA
TGGGTATCTTTTACCCGTTCGTGAACGACAAAAAAGTTAAGCTGGTTGGCGTTGAGGCTGGTGGTAAAGG
CCTGGAATCTGGTAAGCATTCCGCTAGCCTGAACGCAGGTCAGGTTGGTGTGTTTCATGGCATGCTGTCC
TACTTTCTGCAGGACGAAGAAGGTCAGATCAAACCAACCCACTCCATCGCACCAGGTCTGGATTATCCAG
GTGTTGGTCCAGAACACGCTTACCTGAAAAAAATTCAGCGTGCTGAATACGTGACAGTAACCGATGAAGA
AGCACTGAAAGCGTTCCATGAACTGAGCCGTACCGAAGGTATCATCCCAGCTCTGGAATCTGCGCATGCT
GTGGCTTACGCTATGAAACTGGCTAAGGAAATGTCTCGTGATGAGATCATCATCGTAAACCTGTCTGGTC
GTGGTGACAAAGACCTGGATATTGTCCTGAAAGTGTCTGGCAACGTGCTCGAGCACCACCACCACCACC
ACTGA

Translation
MWFGEFGGQYVPETLIEPLKELEKAYKRFKDDEEFNRQLNYYLKTWAGRPTPLYYAKRLTEKIGGAKIYLKRE
DLVHGGAHATNNAIGQALLAKFMGKTRLIAETGAGQHGVATAMAGALLGMKVDIYMGAEDVERQKMNVFRM
KLLGANVIPVNSGSRTLKDAINEALRDWVATFEYTHYLIGSVVGPHPYPTIVRDFQSVIGREAKAQILEAEGQLP
DVIVACVGGGSNAMGIFYPFVNDKKVKLVGVEAGGKGLESGKHSASLNAGQVGVFHGMLSYFLQDEEGQIKP
THSIAPGLDYPGVGPEHAYLKKIQRAEYVTVTDEEALKAFHELSRTEGIIPALESAHAVAYAMKLAKEMSRDEIII
VNLSGRGDKDLDIVLKVSGNVLEHHHHHH-

TrpBK82AHistag

TrpB_T292S_E17G_F95L_I16V_V384A_L161A_L91P_V173E_F274L (TrpB^{7E6})

DNA sequence
ATGTGGTTCGGTGAATTTGGTGGTCAGTACGTGCCAGAAACGCTGGTAGGACCCCTGAAAGAGCTGGAA
AAAGCTTACAAACGTTTCAAAGATGACGAAGAGTTCAATCGTCAGCTGAATTACTACCTGAAAACCTGGGC
AGGTCGTCCAACCCCACTGTACTACGCAAAACGCCTGACTGAAAAAATCGGTGGTGCTAAAATATACCTG
AAACGTGAAGACCTGGTTCACGGTGGTGCACACAAGACCAACAACGCCATCGGTCAGGCACCGCTGGCA
AAGCTCATGGGTAAAACTCGTCTGATCGCTGAGACCGGTGCTGGTCAGCACGGCGTAGCGACTGCAATG
GCTGGTGCACTGCTGGGCATGAAAGTGGACATTTACATGGGTGCTGAGGACGTAGAACGTCAGAAAATG
AACGTATTCCGTATGAAGCTGCTGGGTGCAAACGTAATTCCAGTTAACTCCGGTTCTCGCACCGCGAAAG
ACGCAATCAACGAGGCTCTGCGTGATTGGGAAGCTACTTTTGAATACACCCACTACCTAATCGGTTCCGT
GGTCGGTCCACATCCGTATCCGACCATCGTTCGTGATTTTCAGTCTGTTATCGGTCGTGAGGCTAAAGCG
CAGATCCTGGAGGCTGAAGGTCAGCTGCCAGATGTAATCGTTGCTTGTGTTGGTGGTGGCTCTAACGCG
ATGGGTATCTTTTACCCGTTCGTGAACGACAAAAAAGTTAAGCTGGTTGGCGTTGAGGCTGGTGGTAAAG
GCCTGGAATCTGGTAAGCATTCCGCTAGCCTGAACGCAGGTCAGGTTGGTGTGTTGCATGGCATGCTGT
CCTACTTTCTGCAGGACGAAGAAGGTCAGATCAAACCAAGCCACTCCATCGCACCAGGTCTGGATTATCC
AGGTGTTGGTCCAGAACACGCTTACCTGAAAAAAATTCAGCGTGCTGAATACGTGACAGTAACCGATGAA
GAAGCACTGAAAGCGTTCCATGAACTGAGCCGTACCGAAGGTATCATCCCAGCTCTGGAATCTGCGCAT
GCTGTGGCTTACGCTATGAAACTGGCTAAGGAAATGTCTCGTGATGAGATCATCATCGTAAACCTGTCTG
GTCGTGGTGACAAAGACCTGGATATTGTCCTGAAAGCATCTGGCAACGTGCTCGAGCACCACCACCACC
ACCACTGA

Translation
MWFGEFGGQYVPETLVGPLKELEKAYKRFKDDEEFNRQLNYYLKTWAGRPTPLYYAKRLTEKIGGAKIYLKRE
DLVHGGAHKTNNAIGQAPLAKLMGKTRLIAETGAGQHGVATAMAGALLGMKVDIYMGAEDVERQKMNVFRM
KLLGANVIPVNSGSRTAKDAINEALRDWEATFEYTHYLIGSVVGPHPYPTIVRDFQSVIGREAKAQILEAEGQLP
DVIVACVGGGSNAMGIFYPFVNDKKVKLVGVEAGGKGLESGKHSASLNAGQVGVLHGMLSYFLQDEEGQIKP
SHSIAPGLDYPGVGPEHAYLKKIQRAEYVTVTDEEALKAFHELSRTEGIIPALESAHAVAYAMKLAKEMSRDEIII
VNLSGRGDKDLDIVLKASGNVLEHHHHHH-

TrpB7E6Histag
